# Clinical Correlation Between Overactive Bladder and Allergy in Children

**DOI:** 10.3389/fped.2021.813161

**Published:** 2022-01-14

**Authors:** Lu Yin, Zhou Zhang, Yue Zheng, Ling Hou, Cheng-Guang Zhao, Xiu-Li Wang, Kai-Lei Jiang, Yue Du

**Affiliations:** Department of Pediatrics, Shengjing Hospital of China Medical University, Shenyang, China

**Keywords:** overactive bladder syndrome, allergy, children, desloratadine, correlation

## Abstract

**Background:**

The International Children's Continence Society defines overactive bladder (OAB) as a clinical syndrome characterized by urgency of urination usually accompanied by frequent urination and nocturia symptoms. This study aims to explore the correlation between overactive bladder (OAB) and allergy in children.

**Method:**

The clinical characteristics of 918 patients diagnosed with OAB from January 2020 to March 2021 were retrospectively analyzed. Risk factors for OAB were analyzed using logistic regression analysis, and the effect of desloratadine in the treatment of OAB was evaluated.

**Results:**

The incidence of allergic cough or allergic rhinitis in the mild OAB group was higher than the moderate–severe group. Urodynamics demonstrated that the proportion of patients with a sensitive bladder in the overactive detrusor group was significantly higher than the non-overactive detrusor group. The effective rate of treatment of OAB in patients complicated with allergies and taking desloratadine was 90.14%, which was significantly higher than in patients who were not taking desloratadine, and blood IgE level was a risk factor of ineffective treatment with desloratadine.

**Conclusion:**

OAB is correlated with allergies in children, and desloratadine can effectively improve OAB symptoms.

## Introduction

The International Children's Continence Society defines overactive bladder (OAB) as a clinical syndrome characterized by urgency of urination usually accompanied by frequent urination and nocturia symptoms, with or without urgent urinary incontinence, without urinary tract infection or other definite pathological changes (1). OAB is one of the most common lower urinary tract symptoms in children. In 2002, the incidence of OAB in children in Japan was ~17.8% (2); in 2009, the incidence among children aged 5–13 y in the Republic of Korea was 16.59% (3); and in 2018, the incidence in China was 9.01% (4), according to the cross sectional OAB study in five provinces of mainland China. The etiology of OAB is not very clear, and it may be related to detrusor instability, bladder hypersensitivity, urethral and pelvic floor muscle dysfunction, and other reasons, such as abnormal mental behavior and hormone metabolism disorder. The pathogenesis of OAB in children is more complex than in adults and seriously affects the quality of life of children and families. At present, there is a lack of relatively mature diagnostic and treatment norms for OAB, and the clinical treatment methods for children are limited.

One of our previous studies revealed that children with OAB had other common allergic symptoms, and the effective rate of treatment for OAB by avoiding allergens combined with antihistamine treatment was 96.1%, suggesting that allergic factors are involved in the pathogenesis of OAB (5). Therefore, we expanded the sample size and retrospectively analyzed the risk factors for children with OAB, evaluated the effectiveness of antihistamines in the treatment of OAB and the risk factors for ineffective treatment in patients with allergies and urodynamics in children with OAB in order to improve the understanding of the pathogenesis of OAB in children and provide new treatment ideas for children with OAB complicated with allergies.

## Information and Methods

### Subjects

The subjects were children diagnosed with OAB in the Pediatric Nephrology Department and the Pediatric Dysuria Clinic of the Shengjing Hospital of China Medical University from January 2020 to March 2021. According to the Overactive Bladder Symptom Score (OABSS) criteria (6), the children who met the diagnostic criteria for OAB were divided into the mild and moderate–severe OAB groups. Exclusion criteria: (1) children with urinary tract infections, stones, tumors, diabetes mellitus, diabetes insipidus, or other organic diseases; (2) children complicated with other chronic diseases or an oral drug history; and (3) children with incomplete case data. The studies involving human participants were reviewed and approved by the ethics committee of Shengjing Hospital of China Medical University. Written informed consent to participate in this study was provided by the participants' legal guardian.

Patients without OAB undergoing a physical examination in the pediatric development clinic during the same period were enrolled as the control group.

If a child had suffered from any of the following, they were considered to have an allergy: (1) eczema, (2) urticaria, (3) allergic cough or allergic rhinitis, (4) skin pruritus (including eye rubbing), or (5) blood IgE level higher than the reference value for children of the same age.

### Methods

(1) Follow up and curative efficacy evaluation: All patients with OAB were followed up for 4 weeks after treatment. Overactive bladder symptom score (OABSS) was calculated twice: at enrollment (baseline) and after 4 weeks of treatment. If the OABSS decreased by ≥3 points when compared with the baseline level or the total OABSS was <3 points after 4 weeks, the treatment was considered effective.

(2) Treatment methods: All the patients were placed on a low protein diet, in which the consumption of sugar and other stimulating foods, e.g., carbonated drinks, was reduced, and urination training was conducted. If the families of the patients with OAB complicated with allergies gave consent, the patients were given desloratadine orally once a day in the following dosages: children aged 1–5 y, 1.25 mg; 6–11 y, 2.5 mg; and ≥12 y, 5 mg.

(3) The medical history data were compared between the control and OAB groups, including age; gender; history of eczema, urticaria, allergic rhinitis or allergic cough, skin pruritus (including eye rubbing action), and constipation; and total blood IgE levels, and the risk factors for OAB were analyzed.

(4) The clinical characteristics of the patients with OAB were analyzed, and their urodynamics were summarized. According to the OABSS, the OAB group was further divided into the mild and moderate–severe groups, and the clinical data were compared between the groups.

(5) For patients with OAB complicated with allergies, the efficacy of desloratadine was evaluated 4 weeks after enrollment.

(6) The differences in related factors between the effective and ineffective desloratadine treatment groups were compared, and the risk factors for ineffective desloratadine treatment were analyzed using regression analysis.

### Statistical Methods

Data were analyzed using the statistical software SPSS 21.0. *P* < 0.05 was considered statistically significant. Normally distributed continuous variables were expressed as mean ± standard deviation (x ± SD), and non-normally distributed continuous variables were expressed as the median (interquartile range). Categorical variables were expressed as percentages (%). Continuous variables were compared between groups using the *t*-test or Mann–Whitney test, and categorical variables were compared using the chi-squared test. Variables with statistical significance in the univariate analysis were the subjected to binary logistic regression analysis.

## Results

### General Data

In this study, a total of 918 patients with OAB were included in the OAB group, including 475 males and 443 females with an average age of 6.32 ± 2.168 y. A total of 1,125 patients were included in the control group, including 599 males and 526 females with an average age of 6.32 ± 2.591 y. There were no significant differences in gender ratio and age between these two groups (*P* > 0.05).

### Analysis of OAB Risk Factors

#### Univariate Analysis

Univariate analyses of the history of eczema, urticaria, allergic cough or allergic rhinitis, skin pruritus (including eye rubbing), constipation, and blood IgE levels were conducted between the OAB and control group, and the results revealed that these were significantly higher in the OAB than in control group (*P* < 0.05; see Table 1).

**Table 1:** Univariate analysis of OAB risk factors.

	**OAB group** **(***N*** = 918)**	**Control group** **(***N*** = 1,125)**	***P*** **-value**
Age (year)^Δ^	6.32 ± 2.168	6.32 ± 2.591	0.985
Gender (male, %)^∧^	475 (51.7%)	599 (53.2%)	0.499
Positive eczema (*n*, %)^∧^	582 (63.4%)	137 (12.2%)	<0.001^*^
Urticaria (*n*, %)^∧^	171 (18.6%)	66 (5.9%)	<0.001^*^
Allergic cough, allergic rhinitis (*n*, %)^∧^	263 (28.6%)	67 (6.0%)	<0.001^*^
Skin pruritus (*n*, %)^∧^	489 (53.3%)	97 (8.6%)	<0.001^*^
Constipation (*n*, %)^∧^	105 (11.4%)	41 (3.6%)	<0.001^*^
Blood IgE level (IU/mL)^°^	45.855 (18.81, 131.33)	21.36 (11.69, 39.16)	<0.001^*^

Δ *Data are expressed as mean ± SD*.

∧ *Data are expressed as n, %*.

° *Data are expressed as median 25–75th percentile*.

*SD, standard deviation; n, number*.

* *means P-value <0.05*.

#### Logistic Regression Analysis of OAB Risk Factors

The abovementioned significant factors were included in the binary logistic regression model, which revealed that the risk factors for OAB in children include eczema (OR = 7.299, 95% CI: 5.649–9.524, *P* < 0.001); urticaria (OR = 1.613, 95% CI: 1.091–2.387, *P* = 0.017); allergic cough or allergic rhinitis (OR = 3.484, 95% CI: 2.445–4.950, *P* < 0.001); skin pruritus, including frequent eye rubbing action (OR = 7.194, 95% CI: 5.376–9.524, *P* < 0.001); constipation (OR = 4.016, 95% CI: 2.500–6.452, *P* < 0.001); and blood IgE (OR = 3.785, 95% CI: 2.920–4.906, *P* < 0.001; see Table 2).

**Table 2:** Binary logistic regression analysis of OAB risk factors.

	**OR**	**95% CI**	***P*** **-value**
Age (year)	0.963	0.916–1.013	0.145
Gender (male)	1.188	0.929–1.519	0.170
Positive eczema (*n*, %)	7.299	5.649–9.524	<0.001^*^
Urticaria (*n*, %)	1.613	1.091–2.387	0.017^*^
Allergic cough, allergic rhinitis (*n*, %)	3.484	2.445–4.950	<0.001^*^
Skin pruritus (*n*, %)	7.194	5.376–9.524	<0.001^*^
Constipation (*n*, %)	4.016	2.500–6.452	<0.001^*^
Log (blood IgE)	3.785	2.920–4.906	<0.001^*^

* *means P-value <0.05*.

### Baseline Clinical Characteristics of Patients With OAB

#### Comparison Between OAB Groups With Different Severities

In the OAB group, the baseline OABSS was 6 (4, 7), with 435 mild, 469 moderate, and 14 severe cases. The patients in the moderate and severe groups were combined to form the moderate–severe group. When compared with the moderate–severe group, the age and OABSS were lower (*P* < 0.05) and the incidence of allergic cough or allergic rhinitis was higher in the mild group (*P* < 0.05; see Table 3).

**Table 3:** Comparison among groups with OAB different severities.

**Group**	**OAB mild** **(***N*** = 435)**	**OAB moderate-severe** **(***N*** = 483)**	***P*** **-value**
Gender (male, %)^∧^	228 (52.41%)	247 (51.14%)	0.699
Age (year)^Δ^	6.12 ± 2.090	6.5 ± 2.223	0.008^*^
OABSS score (Baseline)^°^	4 (4.5)	7 (6.8)	<0.001^*^
Eczema (*n*, %)^∧^	262 (60.23%)	320 (66.25%)	0.059
Urticaria (*n*, %)^∧^	77 (17.70%)	94 (19.46%)	0.494
Allergic cough, allergic rhinitis (*n*, %)^∧^	139 (31.95%)	124 (25.67%)	0.036^*^
Skin pruritus (*n*, %)^∧^	221 (50.80%)	268 (55.48%)	0.156
Constipation (*n*, %)^∧^	51 (11.72%)	54 (11.18%)	0.796
IgE level (IU/mL)^°^	41.43 (18.29,120.20)	52.72 (19.98,145.70)	0.059

Δ *Data are expressed as mean ± SD*.

∧ *Data are expressed as n, %*.

° *Data are expressed as median 25–75th percentile*.

*SD, standard deviation; n, number*.

* *means P-value <0.05*.

#### OAB Urodynamic Results

Urodynamic examination was carried out on 152 children with OAB. Of these children, 81 (53.29%) had overactive detrusor and bladder sensitivity, 20 (13.15%) only had overactive detrusor, 19 (12.5%) only had bladder sensitivity, and 32 (21.05%) had normal results. The patients were divided into two groups according to the urodynamic results: the overactive detrusor group (*n* = 101) and the non-overactive detrusor group (*n* = 51). The incidence of bladder sensitivity in the overactive detrusor group was 80.19% (*n* = 81), which was significantly higher than in the non-overactive detrusor group (*n* = 19, 37.25%, *P* < 0.001; see Table 4).

**Table 4:** Comparison of bladder sensitivity between detrusor hyperactivity group and non-detrusor hyperactivity group.

**Group**	**Detrusor overactivity group** **(***N*** = 101)**	**Non-detrusor overactivity group** **(***N*** = 51)**	***P-*** **value**
Bladder sensitivity (*N*, %)^∧^	81 (80.19%)	19 (37.25%)	<0.001
Non bladder sensitivity (*N*, %)^∧^	20 (19.80%)	32 (62.74%)	

∧ *Data are expressed as n, %*.

*n, number*.

^*^means P-value <0.05.

### Analysis of the Efficacy of Desloratadine in the Treatment of OAB

#### The Efficacy of Desloratadine in the Treatment of OAB Complicated With Allergies

Of the patients with OAB complicated with allergies, 629 were treated with desloratadine and were followed up after 4 weeks. The treatment was found to be effective in 567 patients (90.14%). In the effective treatment group, there were 249 patients in the mild group and 318 in the moderate–severe group (310 moderate and 8 severe). In the ineffective treatment group, there were 32 patients in the mild group and 30 in the moderate–severe group (30 moderate and 0 severe). The rate of constipation was higher in the effective group than in the ineffective group (11.64 and 3.23%, respectively; *P* = 0.043), and the IgE levels were lower in the effective group than in the ineffective group (59.90 and 95.40, respectively; *P* = 0.012). There were no significant differences in other factors, including age, gender, eczema, urticaria, allergic cough or allergic rhinitis, skin pruritus (including eye rubbing), baseline OABSS, or severity of OAB between the two groups (see Table 5).

**Table 5:** Univariate comparison between effective and ineffective desloratadine treatment groups.

	**Effective group** **(***N*** = 567)**	**Ineffective group** **(***N*** = 62)**	***P*** **-value**
Age (year)^Δ^	6.272 ± 2.099	6.081 ± 1.8625	0.49
Gender (male, %)^∧^	284 (50.09%)	32 (51.6%)	0.82
Eczema (*n*, %)^∧^	385 (67.90%)	45 (72.58%)	0.452
Urticaria (*n*, %)^∧^	115 (20.28%)	18 (29.03%)	0.109
Allergic cough, allergic rhinitis (*n*, %)^∧^	181 (31.92%)	25 (40.32%)	0.181
Skin pruritus (*n*, %)^∧^	336 (59.26%)	40 (64.52%)	0.423
Constipation (*n*, %)^∧^	66 (11.64%)	2 (3.23%)	0.043^*^
Blood IgE level (IU/mL)^°^	59.90 (25.66, 145.70)	95.40 (38.36, 253.67)	0.012^*^
OABSS score^°^	6 (5,7)	5 (4,7)	0.175
OAB group			
Mild^∧^	249 (43.9%)	32 (51.6%)	0.247
Moderate-severe^∧^	318 (56.1%)	30 (48.4%)	

Δ *Data are expressed as mean ± SD*.

∧ *Data are expressed as n, %*.

° *Data are expressed as median 25–75th percentile*.

*SD, standard deviation; n, number*.

* *means P-value <0.05*.

#### Analysis of Risk Factors for Ineffective Treatment of OAB Complicated With Allergy Using Desloratadine

Age, gender, constipation, and blood IgE levels were converted into logarithms and introduced into the binary logistic regression model. The results revealed that after logarithmic conversion, the blood IgE level was a risk factor for the ineffective treatment of OAB using desloratadine (OR = 1.883, 95% CI: 1.166–3.039, *P* = 0.010) see Table 6.

**Table 6:** Logistic regression analysis of risk factors for ineffective treatment of OAB with desloratadine.

	**OR**	**95% CI**	***P***
Gender (male, %)	1.171	0.686–2.000	0.563
Age (year)	0.925	0.809–1.056	0.250
Constipation	3.891	0.922–16.393	0.064
Log (blood IgE)	1.883	1.166–3.039	0.010^*^

* *means P-value <0.05*.

#### The Comparison of the Efficacy of Combined and Non-combined Desloratadine in the Treatment of OAB With Allergies

There was a total of 850 patients with allergies in the OAB group. Of these, 221 were treated by being given urination training and being placed on a low-protein diet in which the consumption of sugar and other stimulating foods was reduced, and 629 were treated with desloratadine in addition to the above treatment measures. When compared with the non-desloratadine group, the skin pruritus (including eye rubbing), IgE level, and baseline OABSS were significantly higher in the desloratadine group. The curative effect was evaluated after 4 weeks of treatment, and the effective rate was 90.14% in the desloratadine group and 84.16% in the non-desloratadine group (*P* = 0.016; see Table 7).

**Table 7:** Comparison of related factors and efficacies of treatment with and without desloratadine.

	**Desloratadine group** **(***N*** = 629)**	**Non-desloratadine group** **(***N*** = 221)**	***P***
Age (year)^Δ^	6.25 ± 2.076	6.25 ± 2.165	0.966
Gender (male, %)^∧^	316 (50.2%)	117 (52.9%)	0.489
Eczema (*n*, %)^∧^	430 (68.4%)	152 (68.8%)	0.909
Urticaria (*n*, %)^∧^	133 (21.1%)	38 (17.2%)	0.208
Allergic cough, allergic rhinitis (*n*, %)^∧^	206 (32.8%)	57 (25.8%)	0.054
Skin pruritus (*n*, %)^∧^	376 (59.8%)	113 (51.1%)	0.025^*^
Constipation (*n*, %)^∧^	68 (10.8%)	30 (13.6%)	0.268
Blood IgE level (IU/mL)^°^	61.90 (26.66, 152.30)	29.59 (10.57, 100.08)	<0.001^*^
OAB group (Baseline)			0.034^*^
Mild^∧^	281 (44.7%)	117 (52.9%)	
Moderate-severe^∧^	348 (55.3%)	104 (47.1%)	
OABSS score (Baseline)^°^	6 (4, 7)	5 (4, 7)	0.024^*^
**Curative effect**			
Effective^∧^	567 (90.14%)	186 (84.16%)	0.016^*^
Ineffective^∧^	62 (9.85%)	35 (15.83%)	

Δ *Data are expressed as mean ± SD*.

∧ *Data are expressed as n, %*.

° *Data are expressed as median 25–75th percentile*.

*SD, standard deviation; n, number*.

* *means P-value <0.05*.

## Discussion

The incidence of OAB in children ranges from 1.5 to 36.4%, and the peak incidence occurs at 5–7 y and is higher in males than in females (7, 8). In this study, a logistic regression analysis of the risk factors for OAB revealed that allergic manifestations, including eczema, urticaria, allergic cough or allergic rhinitis, skin pruritus (including eye rubbing), and elevated blood IgE levels, were independent risk factors for OAB in children. This study revealed that the age of the patients in the mild OAB group was significantly lower than in the moderate–severe OAB group (*P* < 0.05). Studies have revealed that the incidence of OAB decreases with age, i.e., some OAB symptoms exhibit spontaneous remission (4, 7, 8). However, for children with OAB with severe symptoms and high scores, this is often not the case. Therefore, the average age of the patients in the moderate–severe OAB group is higher than in the mild OAB group.

The exact cause of OAB in children is unclear and may include myogenic theory, neurogenic theory, and abnormal urothelial mediators acting on the smooth muscle/afferent nerves (9). A previous clinical study in our department revealed that children with OAB are often complicated with allergic manifestations, such as eczema, urticaria, skin allergies after mosquito bites, and allergic rhinitis. Soyer et al. (10) revealed that the incidence of urgent and frequent urination was higher in asthmatic children. A cross-sectional cohort study involving 101,848 adult males from South Korea demonstrated that asthma was associated with moderate and severe lower urinary tract symptoms (11).

The results of an experimental study revealed that mast cells could be seen in the urothelium, lamina propria, and smooth muscle layer of the bladder wall; mast cell activation caused histamine release; and the binding of histamine to bladder wall receptors could cause inflammation and bladder allergy (12). In patients with OAB, mast cells in the bladder lamina propria increase, which causes inflammation by releasing inflammatory mediators (13). In sensitized animals, bladder exposure to ovalbumin can lead to an increase in micturition frequency and a decrease in micturition pressure and volume. Therefore, the exposure of bladder mucosa to bladder-sensitized substances could cause micturition changes consistent with the clinical symptoms of “urgent urination and frequent urination” (14, 15). *In vitro* bladder tests revealed that histamine could increase the baseline tension of the lamina propria and detrusor and increase the frequency and amplitude of spontaneous contraction through the H1 receptor (16), and they also found that muscarinic receptors were not involved in this process. This study suggests that the H1 receptor can be used as a potential target for the treatment of OAB and other bladder contraction disorders in the future. As an important neurotransmitter, histamine could also activate the excitatory glutamine signaling pathway and regulate the excitability of sympathetic and parasympathetic nerves in order to regulate lower urinary tract function at the spinal cord level (17). Histamine can also increase the sensitivity of afferent nerves to the bladder through the H1 receptor, resulting in bladder allergy and excessive contraction (18). The abovementioned pathological changes can lead to the occurrence of OAB. Therefore, clinical studies and animal experiments support the hypothesis that chronic allergic inflammation may be involved in the pathogenesis of OAB.

Studies have confirmed that allergies are related to the pathogenesis of chronic cystitis, such as interstitial and eosinophilic cystitis, and antihistamine antagonists, especially antihistamine H1 receptor antagonists, can be used to treat these diseases. Desloratadine is a non-sedating, long-acting tricyclic antihistamine that can selectively antagonize the peripheral histamine H1 receptor and has a low incidence of sedation in the central nervous system and cardiotoxic side effects. The previous clinical study in our department revealed that for patients with daytime frequent urination and an allergic constitution, an allergen avoidance diet combined with antihistamine treatment can significantly improve symptoms without obvious side effects (19).

In this study, 629 children with OAB complicated with allergies were treated with diet control, behavioral training, and oral desloratadine, and after 4 weeks, the effective remission rate of OABSS was 90.14%, which was significantly higher than similar patients who were not treated with desloratadine (84.16%) over the same period (*P* = 0.016). Blood IgE levels were shown to be a risk factor for the ineffective treatment of OAB with desloratadine (OR = 1.883, 95% CI: 1.166–3.039, *P* = 0.010). In patients with specific dermatitis, high levels of IgE are usually associated with disease severity (20, 21). Patients with asthma of long duration who have poor lung functions have high IgE levels (22).

The results of this study also revealed that the serum IgE levels in the moderate–severe group were higher than in the mild group. It is speculated that high IgE levels indicate a prolonged duration of OAB and severe OAB. Therefore, the affected sites extend from the bladder mucosa to the smooth muscle, which may lead to treatment failure. In addition, IgE can induce mast cells to release histamine and cause the release of other mediators, such as leukotriene, bradykinin, and prostacyclin. Therefore, the effect of antihistamine for only 4 weeks was poor, and it may be necessary to extend the treatment time or combine desloratadine with other antiallergy drugs to improve the curative effect.

The typical urodynamic change in patients with OAB is the involuntary contraction of the detrusor during urine storage. Many studies have shown that the activation and upregulation of the sensitivity of the sensory nerves play an important role in the pathogenesis of OAB, and nerve fibers innervating the urothelium and subepithelial tissue are activated, resulting in frequent and urgent urination (23). In this study, the most common urodynamic result was detrusor overactivity combined with bladder hypersensitivity, accounting for 53.29%. The proportion of patients with bladder hypersensitivity was significantly higher in the overactive detrusor group than in the non-overactive detrusor group (80.19 and 37.25%, respectively; *P* < 0.001). Lee et al. (9) revealed that the bladder of patients with OAB was both hyperactive and hypersensitive, which is consistent with the results of this study. Bladder hypersensitivity may be caused by allergic inflammatory mediators in the submucosal blood supply acting on mucosal epithelial cells, and the involuntary contraction of the detrusor may be caused by the action of allergic inflammatory mediators in the blood supply on detrusor cells. Abnormalities in these can lead to bladder mucosal hypersensitivity and involuntary detrusor contraction and the corresponding lower urinary tract symptoms, i.e., frequent urination, urgent urination, and dripping.

In this study, the clinical data of children with OAB were retrospectively analyzed, and the results revealed that the risk factors for OAB in children were eczema, urticaria, allergic cough or allergic rhinitis, skin pruritus (including frequent eye rubbing), constipation, and increased blood IgE levels. Most children with detrusor overactivity were complicated with bladder hypersensitivity. Antihistamines were effective in the treatment of patients with OAB complicated with allergies. Therefore, it can be assumed that OAB is correlated with allergies in children, which offers a new idea for the exploration of the pathogenesis and treatment of OAB in children.

This study was a retrospective study. Prospective studies should be designed in the future to reduce bias and the other negative effects of retrospective studies.

## Data Availability Statement

The original contributions presented in the study are included in the article/supplementary material, further inquiries can be directed to the corresponding author/s.

## Ethics Statement

The studies involving human participants were reviewed and approved by the Ethics Committee of Shengjing Hospital of China Medical University. Written informed consent to participate in this study was provided by the participants' legal guardian/next of kin.

## Author Contributions

YD and LY: conception and design of the research. ZZ and YZ: acquisition of data. LH and X-LW: analysis and interpretation of the data. LY and K-LJ: statistical analysis. YD: obtaining financing. LY: writing of the manuscript. C-GZ: critical revision of the manuscript for intellectual content. All authors read and approved the final draft.

## Conflict of Interest

The authors declare that the research was conducted in the absence of any commercial or financial relationships that could be construed as a potential conflict of interest.

## Publisher's Note

All claims expressed in this article are solely those of the authors and do not necessarily represent those of their affiliated organizations, or those of the publisher, the editors and the reviewers. Any product that may be evaluated in this article, or claim that may be made by its manufacturer, is not guaranteed or endorsed by the publisher.
